# Are health sciences students who sit at the back of the lecture hall not motivated?

**DOI:** 10.1371/journal.pone.0174947

**Published:** 2017-03-31

**Authors:** Sébastien Uffler, Jean-Claude Bartier, Thierry Pelaccia

**Affiliations:** 1 Prehospital Emergency Care Service (SAMU 67), Strasbourg University Hospital, Strasbourg, France; 2 Centre for Emergency Care Teaching (CESU 67), Strasbourg University Hospital, Strasbourg, France; 3 Emergency Departments Network in Alsace (RESURAL), Strasbourg, France; 4 Centre for Training and Research in Health Sciences Education (CFR-PS), Faculty of Medicine, University of Strasbourg, Strasbourg, France; University of Westminster, UNITED KINGDOM

## Abstract

**Objectives:**

Motivation is a crucial determinant in learning and performance. It would therefore be advantageous for teachers to use strategies intended to have a positive effect on their students' motivation. With this in mind, the first thing to do is to identify students with motivation problems, which can be a complex exercise when there are large groups. We wanted to explore whether the place chosen by health sciences students in a classroom or lecture hall showed any correlation with their motivation.

**Methods:**

We carried out a multicentre, prospective, observational study of 596 health sciences students in 9 training institutes. The students filled in a self-administered questionnaire to measure the different components of their motivation to take part in a mandatory lesson. These components were correlated with the row in which they sat in a classroom or lecture hall, when they had a free choice of where to sit.

**Results:**

Apart from extrinsic motivation, all the components of motivation for the health sciences students recruited were significantly correlated with the row. The further the students were from the first row, the less they were motivated.

**Conclusion:**

In accordance with teachers' views, the level of motivation of the students was less the further their position in a classroom or lecture hall was from the first row. A student's position in the classroom could provide a useful indicator for teachers looking to target their motivational strategies for students with potential motivation problems in the environment, where identifying student motivation levels is impossible.

## Introduction

Motivation is known to be one of the most important factors for success in learning and achievement [1]. According to the self-determination theory, level and type of motivation differ from one person to the next [2]. Two types of motivation are thus defined as a routine [3,4]:

- Extrinsic motivation is a kind of motivation which is external to the person concerned, which pushes them to take action, for example by obligation, by constraint or the desire for reward. That’s for instance the case for nursing students who learn their lessons because they consider that getting a good grade is the most satisfying thing for them or whose only objective is not failing their exams.
- Intrinsic motivation defines the motivation to take part in an activity for the pleasure, benefit or satisfaction to be obtained directly from it. That’s for instance the case for nursing students who learn because they like learning new things or who choose course assignments they can learn from, even if they don’t guarantee a good grade.

Intrinsic motivation is higher when the person’s perception of the value of the task (importance the person attributes to a given task), its efficacy (the person’s perception of his/her ability to complete the task successfully) and control (level of autonomy and control perceived by the person when carrying out the task) is high [1].

Research since the early 20^th^ century has shown that students with an intrinsic motivational profile work harder at tasks linked to learning and persevere in spite of difficulties, use more effective learning strategies and, finally, achieve a higher level of performance [5]. It would therefore be beneficial for teachers to implement strategies which preferentially promote intrinsic types of motivation and the different components with which it is positively correlated. This prospect involves initially identifying students with a motivation problem, which may be complex—or impossible—when they are in large groups.

We have performed a preliminary study intended to explore the observations of 26 teachers in four health sciences schools concerning the links between students’ position in a classroom or lecture hall and their motivation level. 62% of those who answered a self-administered questionnaire judged that the place a student chooses in a classroom is mainly based on his/her motivation to attend the class. These observations may lead teachers to behave in a way which is potentially prejudicial to learning for students sitting at the back of the class, by paying less attention to them, owing to an effect known as the “Pygmalion” effect. This effect was documented at the end of the 1960s by Rosenthal and Jacobson who demonstrated the impact of teachers’ observations about their students concerning the students’ intellectual ability and performance [6].

In this work, we wanted to determine whether or not these observations about motivation match reality, by answering the following question: is a health sciences student’s position in a classroom an indicator of his/her motivation?

## Materials and methods

### Study design and study population

A multicentre, prospective, observational study was carried out between February 2012 and May 2014, involving 596 health sciences students in 9 health sciences schools in Alsace (France). They were made up of 477 nursing students, 40 nurse-anaesthetist students, 39 child-care auxiliary students, 24 obstetric nurse students and 16 health management students. All respondents were over the age of 18. They had not been informed of the exact study objectives. The aim was to avoid influencing their answers, which is always a risk when studying motivation. A cover letter was given to inform them that their participation was entirely voluntary. Participants were given the choice not to fill out the questionnaire if they didn’t want to participate to the study. Written consent was not required. The students were informed that the data would be treated confidentially and the questionnaires destroyed at the end of the study. We also emphasized the fact that all the data would be anonymised and it would not be possible to link the study results to any individual person.

### Questionnaire design

We used the Motivated Strategies for Learning Questionnaire (MSLQ), drafted by Pintrich et al. [7,8]. The reliability and predictive validity of the MSLQ have been assessed and revealed to be strong [8]. Using several subscales, this anonymous questionnaire is used to explore the different components of motivation: intrinsic motivation, extrinsic motivation, perceived task value, perceived self-efficacy and control of learning beliefs. Twenty-six questions were submitted to the study population. The answers were transcribed to a Likert scale graduated from 1 (not at all true of me) to 7 (very true of me). Table 1 shows each of the five motivation components explored, with a sample question for each component. The questionnaire also included an open question intended to explore more broadly the reasons for choice of seat in the classroom or lecture hall: “Why did you sit in that place today?”. The anonymous questionnaire was self-administered to the participants at the start of a lecture held in a classroom or lecture hall in which students could choose to sit where they wanted. One lesson for each class in each institute was targeted randomly by the researchers. Two exclusion criteria had been defined concerning the choice of the lesson:

- A lecture given by a teacher known to the students, because of the possible influence of prior knowledge about the teacher on the choice of seat.
- A lecture given somewhere other than a classroom or lecture hall.

**Table 1:** Components of motivation explored by the Motivated Strategies for Learning Questionnaire and sample question for each component.

Component of motivation explored	Example of affirmation used to explore this component
Intrinsic motivation	The most satisfying thing for me in this course is trying to understand the content as thoroughly as possible
Extrinsic motivation	Getting a good grade in this class is the most satisfying thing for me right now
Perceived task value	I think the course material in this class is useful for me to learn
Perceived self-efficacy	I’m confident I can understand the most complex material presented by the instructor in this course
Control of learning beliefs	If I try hard enough, then I will understand the course material

The questionnaire was distributed to each student in each class before the start of the lesson. Respondents were verbally instructed to note on the questionnaire the number of the row identified by the researcher in charge of collecting the data (from 1 to 9). The student had ten minutes in which to fill in the questionnaire, which was then immediately collected. Participants were given the choice not to fill out the questionnaire if they didn't want to participate to the study.

### Questionnaire analysis

We strictly followed the recommendations made by the authors of the Motivated Strategies for Learning Questionnaire (MSLQ), to analyze data [7]. As highlighted by Pintrich et al., “scales are constructed by taking the mean of the items that make up that scale. For example […], an individual’s score for intrinsic goal orientation would be computed by summing the four items and taking the average”. For each component of motivation, we calculated the mean obtained by all the students in a single row, then the coefficient of correlation between this mean and the row. More accurately, the relationship between the row occupied by the student and the Likert score was estimated using a linear model. The adequacy to the model was measured by a multiple linear regression coefficient (r-squared). We checked that the coefficients were significantly different from zero using Fisher and Student’s tests. The level of statistical significance was set at p<0.05. Data analysis was performed by a statistician using R software (version 3.1.0). Full data are available as Supporting information (S1 Data).

Our work has been approved by the ethics committee of the faculty of medicine of Strasbourg.

## Results

Five hundred and ninety-three students were included in the study. Three subjects were excluded owing to the presence of aberrant values suggesting a random response to the questions asked. Nine groups of students were identified, each group representing one row. We found a high correlation between the row and all motivation components (Intrinsic motivation: r^2^ = 0,73; p = 0,003 –Perceived task value: r^2^ = 0,84; p = 0,0004 –Perceived self-efficacy: r^2^ = 0,63; p = 0,01 –Control of learning beliefs: r^2^ = 0,48; p = 0,038), except extrinsic motivation (r^2^ = 0,20; p = 0,22). This means that the further the student was from row 1, the lower his/her motivation.

Concerning the open question intended to identify the reasons specifically given by the students to justify their choice of seat, we grouped them into categories represented by a decreasing order of frequency in S1 Graph.

## Discussion

We showed that there is a significant correlational link between the positioning of health sciences students in the classroom and their level of motivation, no matter what motivation component is involved, except for extrinsic motivation. In accordance with popular observations and, probably, those of a majority of teachers, the level of motivation of students at the back of the class is lower than that of students sitting in the front rows.

### An opportunity to identify students with motivation problems

These results are likely to reinforce the Pygmalion effect, because once the teacher actually knows that the most motivated students sit in the front rows, the teaching methods and interactions with students at the back of the class may be affected.

On the other hand, the results of this research could enable teachers to use a more rational approach to the links between the students’ position in the classroom and their motivation to pay attention to the lesson. The aim is to help teachers to take the “motivation” factor into account when designing their teaching strategies. We have previously emphasized the crucial nature of this component in the process of learning and performance. However, an individual’s level of motivation is complex to evaluate, particularly on the scale of a large group. This is all the more true in situations in which the teacher is meeting the students for the first time. The student’s position in the classroom could therefore act as an indicator of motivation level, enabling the teacher to particularly identify students with a potentially lower motivation level.

### Targeted motivational strategies

This observation could be taken into account, discussed and used during interviews with the student or when supervising individual work. It could also be used with the prospect of implementing teaching and evaluation strategies which would act positively on student motivation, so that the teacher could particularly target students at the back of the class.

These strategies mainly aim at enhancing perceived task value, perceived self-efficacy and control of learning beliefs. The higher these three components of a student’s motivation, the higher his or her motivation. Based upon previous publications, Table 2 describes some relevant strategies in order to reach this goal, for each component of the motivation [9].

**Table 2:** Various strategies to enhance the three components of a student’s motivation.

Explore expectations and projects	**→** Improve perceived task value
Take time to explain the purpose of the material taught	
Use challenging activities (nor too easy nor too difficult)	
Promote problem-solving approaches	
Make links between theory and practice	
Use motivating assessment strategies that promote success	**→** Improve perceived self-efficacy
Support failures by providing appropriate feedback	
Encourage progress and persistence	
Give significant choices to students	**→** Improve control of learning beliefs

### Other factors influencing the choice of seat

It should be stated that we only established correlational links and not causality links. Other factors may influence the choice of seat. These were partly explored in our study. This concerns particularly the “practical” nature of the seat chosen, for example providing a better view or where it is easier to hear the teacher, or be able to plug in a laptop.

### Strengths and weaknesses of the study

Our study was carried out on a large population taken from several health sectors, to promote generalization.

According to the recommendations made by the authors of the MSLQ, we analyzed the data considering the variables as being quantitative [7]. It should, however, be noted that Likert values, while they are numeric, are only a code and not a metric. Therefore, they should be considered as qualitative variables (more specifically, ordinal variables). As a consequence, performing summary statistics such as the mean and median is subject to criticism by some researchers. Other researchers, notably in the field of medical education, believe that this way of processing data may be acceptable [10]. We decided to strictly follow the recommendations made by the authors of the MSLQ to analyze data [7], like other researchers do when using this questionnaire [1,11–14]. From a statistical viewpoint, this should, however, be considered as a limitation.

In addition, although the predictive validity and reliability of the MSLQ have been evaluated and shown to be high [8], we had to translate the items into French. The translated questionnaire was not tested in preliminary studies. Furthermore, we used an exclusively quantitative approach. Carrying out semi-directed interviews would help gain an in-depth understanding of the factors motivating a student’s choice of seat in a classroom or lecture hall, and the influence of the motivation to attend the lesson in question.

To our knowledge, no scientific study has so far explored the possible existence of links between the motivation and positions chosen by health sciences students in a classroom. Students sitting at the back of the class are less motivated than others. This discovery, which seems coherent with teachers’ observations, might be linked to a positive or negative impact, depending on whether the teacher manages to integrate these results into the implementation of strategies acting positively on motivation. Further work could allow us to clarify the respective influences of the various factors mentioned above in the choice of seat by a student, and to specify the part played by motivation in this choice.

## Supporting information

S1 Graph Reasons given by the students in answer to an open question exploring the reasons for their choice of seat in the classroom or lecture hall (in decreasing order of frequency). (DOCX)

S1 Data Data analyzed in the study. (XLS)
