# Supplementary material for: Are health sciences students who sit at the back of the lecture hall not motivated?
Source: PLoS One. 2017 Mar 31;12(3):e0174947. doi: 10.1371/journal.pone.0174947 (PMC5376317; doi:10.1371/journal.pone.0174947)
Supplement: S1 Graph — (DOCX) [file pone.0174947.s001.docx]

**Graph 1: Reasons given by the students in answer to an open question exploring the reasons for their choice of seat in the classroom or lecture hall (in decreasing order of frequency)**


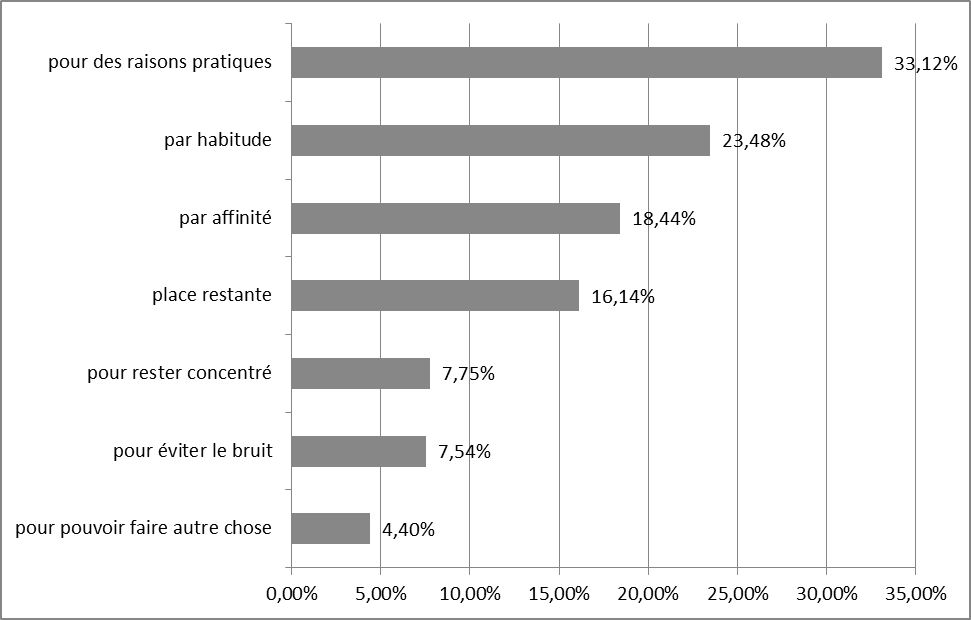


For practical reasons

Through habit

Through affinity

Remaining seat

To stay concentrated

To avoid noise

To be able to do something else
